# Free-Standing Boron Doped Diamond Slot Electrodes for UV–Visible Spectroelectrochemistry: Electrochemical Advanced Oxidation and Metal Ion Reduction

**DOI:** 10.1021/acselectrochem.5c00085

**Published:** 2025-05-12

**Authors:** Anjali John, Anna Dettlaff, Joshua J. Tully, Julie V. Macpherson

**Affiliations:** † Department of Chemistry, University of Warwick, Coventry, United Kingdom CV4 7AL; ‡ Faculty of Chemistry, Gdańsk University of Technology, Narutowicza 11/12, 80-233 Gdańsk, Poland

**Keywords:** UV-Vis spectroelectrochemistry, boron doped diamond (BDD), free-standing BDD, laser-micromachining, electrochemical advanced oxidation, brilliant blue dye, electroreduction, palladium electrodeposition

## Abstract

Boron doped diamond (BDD) has numerous advantages as an electrode material such as having a wide aqueous solvent window, water oxidation, which is thought to produce weakly adsorbed hydroxyl radicals, low background currents, and high electrochemical stability. While BDD has received interest as an optically transparent electrode for combined UV-Vis electrochemical measurements, there are no studies which use it in applications which capitalize significantly on the properties of BDD. In this paper, we describe the use of a BDD spectroelectrochemical (SEC) electrode, BDD_SEC_, fabricated from free-standing BDD (400 μm thickness) and containing laser-micromachined slot-shaped holes (360 μm wide). The electrode shows an optical transmittance of 63% within the wavelength range of 200 to 800 nm, which is the highest reported transmittance for a BDD SEC. UV-Vis electrochemical characterization measurements are made using the redox couple Ru­(bipy)_3_^2+/3+^ over a wavelength range that indium tin oxide electrodes struggle to access due to high background absorption in the UV region. Time scales for Ru­(bipy)_3_^2+^ conversion to Ru­(bipy)_3_^3+^ in this setup are ascertained. We demonstrate the first *operando* measurements for removal of a UV-Vis active molecule (brilliant blue) using BDD_SEC_ electrodes under advanced oxidation conditions. From the change in the UV-Vis absorption signal with time, comparative measurements of the removal rate as a function of applied potential can be obtained; specifically rate constants of 0.10 min^–1^ (1.04 V), 0.24 min^–1^ (at 1.39 V), and 0.68 min^–1^ (at 2.22 V) *vs* Ag|AgCl (3 M Cl^-^) are determined for this experimental arrangement. At the highest potential, we propose both direct and indirect oxidation (via production of hydroxyl radicals from water) are possible. As a second application, we demonstrate the viability of the BDD_SEC_ electrode for quantifying metal ion removal rates (via electroreduction) from different solvent systems. Specifically, we consider electrochemical removal of Pd from Pd–acetate in aqueous acid and in a mixed water:acetonitrile solution.

## Graphical abstract

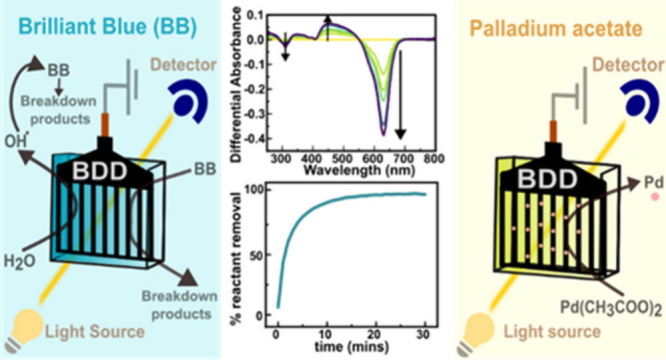

## Introduction

Spectroelectrochemistry (SEC) is a powerful analytical technique which enables operando spectroscopic information during electrochemical reactions.^1,2^ Over the last half-century,^3^ electrochemistry has been integrated with various spectroscopic techniques such as UV–visible (UV-Vis), infrared, and Raman spectroscopy. This has enabled a range of applications including reaction mechanism elucidation, development of electrochromic devices, investigation of compounds of biological interest, sensor development, *etc*.^2^ Among these, UV-Vis SEC is particularly popular, given its applicability to conjugated organic systems, photosynthetic pigments, transition metal complexes, *etc*.^1^ Using UV-Vis SEC electrochemically induced reactant/intermediate/product concentration changes can be monitored via changes in the accompanying UV-Vis electronic transition spectra.^4^

UV-Vis SEC requires optically transparent electrodes (OTEs) that must meet two key criteria: good electrical conductance and sufficient optical transparency. The most commonly used OTEs are indium (or fluorine) tin oxide (ITO)^5^ deposited onto transparent materials such as glass or quartz, thin metal films (typically less than 200 nm in thickness),^6,7^ or metal meshes.^8^ However, the use of these materials can pose some challenges. For example, (i) even though ITO shows an extended aqueous window in the positive direction, compared to metal electrodes,^9^ it has a lack of transparency in the UV region and undergoes chemical degradation at cathodic potentials;^10,11^ (ii) electrocatalytic metal electrodes can dissolve at high anodic potentials; and both materials show (iii) instability in harsh chemical environments and (iv) lower electrical conductance attributed to the thinness of the films.

Polycrystalline boron doped diamond (BDD) has been explored extensively as an electrode material due to its unusual electrochemical and material properties.^12^ These include a wide aqueous solvent window, due to the low electrocatalytic activity, which is thought to favor production of (weakly-adsorbed) hydroxyl radicals (•OH) from water oxidation,^13^ low background currents, electrochemical/chemical stability under a wide range of conditions, resistance to fouling, and mechanical robustness.^12^ As a result, BDD has found use as an electrode in applications as diverse as electrochemical advanced oxidation for pollutant removal and detection of biological analytes at low concentration.^14^ The vast majority of studies use polycrystalline BDD, not single crystal, due to the reduced cost and ease of growth. There has been interest in the use of polycrystalline BDD as an OTE; however, studies in the literature mostly focus on investigating the UV-Vis response of the reactant and product for (quasi)-reversible redox systems.^15^ No studies to date use single crystal BDD as an OTE.

Introducing boron atoms into the diamond lattice to provide enough charge carriers for metallic doping turns the diamond black, with absorption increasing as the wavelength moves from the UV to the IR regions.^16^ To counter this for OTE studies, BDD is often grown as a thin film (0.5–1 μm thick) on a transparent substrate (quartz or undoped silica).^17–19^ In this configuration a maximum transmittance of 60% has been recorded in the visible region (for a boron concentration of 9 × 10^20^ B atoms cm^–3^).^17^ Thin film BDD possesses small grains, resulting in high grain boundary density. Given that sp^2^ carbon predominantly exists within grain boundaries, growing such thin films typically leads to an increased occurrence of sp^2^ carbon. sp^2^ carbon compromises the electrochemical properties of the BDD.^12^ Additionally, thin film BDD is characterized by increased resistance and risk of delamination from the growth substrates at high current densities.^20,21^ Limited studies have employed significantly thicker and consequently lower grain density BDD films.^22,23^ Here the BDD has been grown thick enough, over two orders of magnitude thicker (∼400 μm and thicker), such that it can be removed from the growth substrate and used in this form as an electrode; this material is referred to as “free-standing” BDD. To accommodate the reduced optical transmission due to the thickness increase, a much lower boron concentration is utilized. This moves the electrochemical properties of the BDD into the *p*-type semi-conducting regime.

Given the use of metal meshes as UV-Vis SEC electrodes^8^ there has also been limited work utilizing BDD which contains holes running all the way through the material. This has been achieved by growing the BDD onto a pre-existing Pt mesh^24^ or by laser machining through-holes into free-standing BDD to significantly increase optical transparency.^25–27^ In this configuration, the BDD can always be used at a dopant density high enough to show metal-like conductivity, light can pass freely through the holes, and the machining process enables easy tuning of the through-hole density and geometry. Free-standing BDD also has the advantages that the as-grown face can be grown under conditions which result in minimal sp^2^ carbon,^28^ it does not suffer from delamination issues and can withstand high current densities. With respective to the latter, quantitative studies have shown, using the same free-standing BDD as used herein (in strong electrolytes), a corrosion rate of less than 1 nm h^–1^ at a high current density of 1 A cm^–2^.^29^

To date, there are only a few studies using free-standing BDD through-hole electrodes for SEC.^25–27^ Here we expand the range of *operando* applications of BDD_SEC_ electrodes to those that truly exploit the electrode properties of BDD. Specifically, we explore the use of the BDD_SEC_ electrode to (i) quantify the electrochemical removal rates of the dye, brilliant blue, as a function of applied anodic potential and (ii) explore metal complex removal from different solvent systems via electroreduction. For all studies, a BDD_SEC_ electrode containing slot-shaped holes is utilized.

## Experimental Section

### Solutions

Solutions were prepared using tris­(bipyridine) ruthenium­(II) chloride (Ru­(bipy)_3_Cl_2_, Sigma-Aldrich, UK), potassium nitrate (KNO_3_, ACS reagent, ≥99%, Sigma-Aldrich, UK), hydrochloric acid (HCl, 35%, VWR International), brilliant blue for coloring food (BB FCF) (≥97%, analytical standard, Sigma-Aldrich, UK), palladium acetate (Pd_3_(CH_3_COO)_6_, ≥99.9% trace metal basis, Sigma-Aldrich, UK), tetrabutyl ammonium hexafluorophosphate (TBAPF_6_, 98%, Sigma-Aldrich, UK) and potassium chloride (KCl, ≥99%, Sigma-Aldrich, UK). All chemicals were used as received without further purification. Aqueous solutions were prepared in ultrapure water (>18.2 MΩ cm, Milli-Q, Millipore Corp). Non-aqueous solutions were prepared using dry acetonitrile (MeCN) (Extra Dry over Molecular Sieve, ACROS Organics). Solutions were freshly prepared before each experiment and were performed at ambient temperatures (20 ± 2 °C).

### Electrode Fabrication

BDD_SEC_ electrodes were cut from free-standing electroprocessing (EP) grade (Diafilm^TM^) polycrystalline BDD (Element Six, UK) of 400 μm thickness, metallically doped with a resistivity of ca. 0.5 × 10^–3^ Ω m.^30^ Commercial costs of this material are given in reference 27. The free-standing BDD contains an as-grown, large grain size, low grain density growth face (top growth surface) and a small grain size, high grain density nucleation face (bottom growth surface, which was previously in contact with the growth substrate). The growth face contains low sp^2^ carbon content, due to the large grain sizes, as shown previously by us, using Raman microscopy (polished electrode D in reference 28) and as demonstrated by others (on the same material).^27,28^ The growth face of the as-grown wafer had an arithmetic average roughness (*R*_*a*_) of ∼8 μm, while the nucleation face had an *R*_*a*_ roughness of ∼100 nm.^30^ The outline shape of the BDD_SEC_ electrode was machined using a 355 nm Nd:YAG 34 ns pulse laser micromachining system (E-355H-ATHI-0 system, Oxford Lasers). To produce the slots the laser micromachining trepan system was employed to widen the laser spot diameter to 50 μm in order to make the cut trench wider and remove the need for a kerf on the cuts. This adjustment ensures that the slots machined on the growth and nucleation faces of the BDD electrode were the same size. Electrodes were cut using two passes (internal slots and outline) with a fluence of 760 J cm^–1^ per pass. The BDD_SEC_ electrodes were subjected to an oxidative acid treatment by boiling in a mixture of concentrated sulfuric acid (H_2_SO_4_, >96%, Merck) and potassium nitrate (KNO_3_, 99.97%, Sigma-Aldrich),^31^ to minimize the sp^2^ carbon content in the surface, resulting from laser micromachining.^31^ The contact pad area of the electrode (Figure S1, Supporting Information, SI 1.1) was laser-roughened (fluence = 20 J cm^–2^) to improve the adhesion of the electrical contact, formed by deposition of a trimetal stack of Ti (50 nm)|Pt (50 nm)|Au (200 nm), using a NanoPVD deposition system (Moorfield, UK). After deposition, the contact was annealed at 600 °C for 5 min in a rapid thermal annealer (Solaris 100, Surface Science Integration, USA).

### SEC Cell Design

The BDD_SEC_ comprised 11 through-slots, each of width 360 μm, separated by 260 μm of BDD (Figure 1a). The electrodes were placed in a custom-designed housing and 3D printed in Rigid 10 K resin (FormLabs, USA) to both exclude solution from the electrical contact and allow easy integration with the SEC cell. The lower part of the BDD_SEC_ electrode which contains the slot pattern is a square, 0.5 × 0.5 cm, with rounded corners, which transitions to a thinner rectangle of width 0.2 cm × 0.7 cm length (Figure 1a and Figure S1b). The electrodes were sealed in the housing, and a conductive epoxy (Chemtronics, USA) was used to make electrical contact with a copper wire. A detailed procedure for this step of the electrode fabrication is given in SI 1.1.

**Figure 1:**
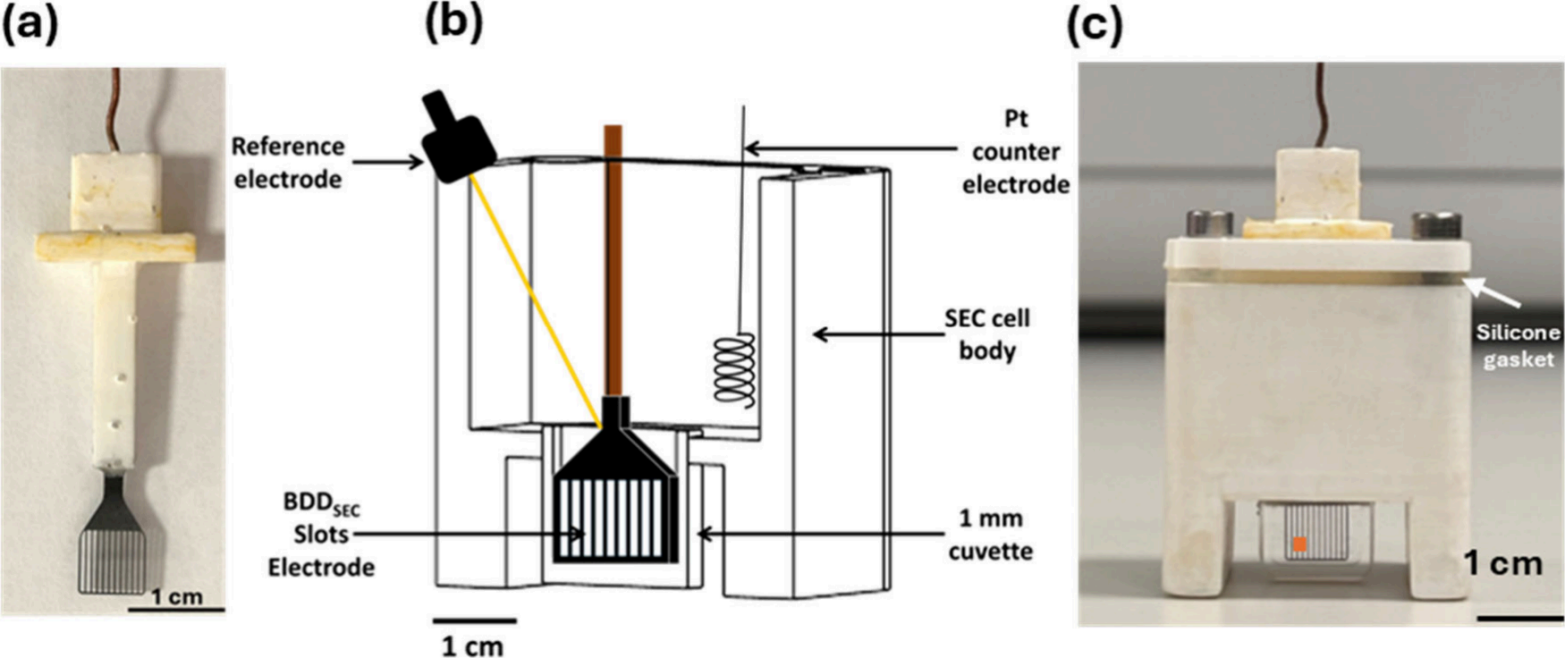
(a) Photograph of electrode housed in 3D printed material, (b) cross-sectional schematic of the SEC cell, and (c) photograph of 3D printed SEC cell (printed in rigid 10 K resin for non-aqueous studies; orange spot corresponds to the UV-vis beam).

Figure 1b shows a cut-through of the SEC cell into which the BDD_SEC_ is placed, along with the reference and counter electrodes. The body of the SEC cell consisted of a headspace (volume of 8 mL) and a commercial 1 mm path length quartz cuvette (volume = 80 μL; Hellma, UK). The BDD_SEC_ electrode, of thickness 400 μm, is placed in the cuvette and occupies the majority of the cuvette as shown in Figures 1b and c. The cuvette and BDD_SEC_ electrodes were oriented perpendicular to the UV-Vis beam of size ∼1.5 mm × 1.0 mm (the orange spot in Figure 1c indicates typical beam position). The headspace was large enough to accommodate both the reference and counter electrodes, as shown in Figure 1b. The counter was placed sufficiently far from the BDD_SEC_ electrode to avoid counter electrolysis products diffusing into the cuvette over the time scale of the measurement. The body of the SEC cell was designed in Fusion 360 (Autodesk, USA) and 3D printed^24^ on a Form 3 stereolithography (SLA) 3D printer, in either clear (for aqueous solutions) or Rigid 10 K (for non-aqueous solutions) resins (Figure 1c), both from FormLabs.^32^ Printed parts were washed and UV and thermally cured according to the manufacturer’s instructions.

3D renders of the SEC cell body can be seen in Figure S2, SI 1.2. The small opening in the base of the headspace through which the cuvette is placed is visible. The cuvette is adhered to the opening by using the same resins utilized for printing the cell. The SEC cell is held in place using screws, on a 3D printed platform (Figure S3 in SI 1.3) inside the UV-Vis spectrophotometer. Screws are also used to secure a cap in place on top of the cell, which acts to minimize the ingress of air. Holes in the cap provide access for the quartz cuvette, reference, counter electrodes, and degassing lines as shown in Figure S2 of SI 1.2. To further minimize air ingress, silicone gaskets, cut using a Cricut maker (Cricut, UK), were used to seal the headspace and the slot through which the BDD_SEC_ electrode is inserted into the cuvette. Blu-tak and O rings were employed to seal the counter electrode and reference electrodes in place, and the BDD_SEC_ electrode was bolted in place to prevent movement during measurement (see Figure S2 in SI 1.2 for a top view of the SEC cell).

### Electrochemical and SEC Measurements

Electrochemical measurements, including cyclic voltammetry (CV) and chronoamperometry, were carried out using a three-electrode configuration in conjunction with an Autolab PGSTAT204 potentiostat (Metrohm, Switzerland). A 1 mm diameter BDD disc sealed in glass (SI 1.4 provides details on the BDD material used and sealing procedures), 2.5 mm diameter Au disc electrode (CH instruments, UK), 3 mm diameter GC disc electrode (CH instruments, UK), and BDD_SEC_ slot electrode were used as the working electrodes. A coiled Pt wire served as the counter electrode. In aqueous solutions, a non-leak Ag|AgCl (3 M Cl^–^, Alvatek, UK) reference electrode was employed. An in-house fabricated Ag|Ag^+^ electrode (filling solution was 10 mM of silver nitrate in 0.1 M TBAPF_6_ in MeCN) was employed as the reference for non-aqueous solutions. The filling solution was changed daily to maintain the potential stability. Pd removal studies were performed under an inert atmosphere of N_2_. To ensure a clean BDD_SEC_ electrode prior to measurement, the electrodes were cycled in 0.5 M H_2_SO_4_ from −2.0 to 2.0 V *vs* Ag|AgCl (3 M Cl^-^) for ten cycles and polished using a small piece of microcloth pad with alumina powder (0.05 μm, Buehler, USA) and ultrapure water. Electrochemical double layer capacitance (EDL), solvent window, and redox mediator tests were conducted to characterize the fabricated BDD_SEC_ slot electrode (see SI 2). The EDL and solvent window measurements indicate that the material does not contain significantly high levels of sp^2^ carbon.

Solution pH was measured using a glass pH probe (Mettler Toledo, UK) in triplicates, and an average was reported. UV-Vis absorption measurements were performed by using a Cary-60 UV-Vis spectrophotometer (Agilent, UK). The absorbance spectra collected under electrochemical control are represented herein as differential spectra (*vide infra*).

## Results and Discussion

### Spectroelectrochemical Characterization of the BDD_SEC_ Slot Electrode

Figure 2a shows an optical image of the BDD_SEC_ slot electrode. Optical transmittance data was recorded over the wavelength range 200 to 800 nm, resulting in an average value of 63.40 ± 0.12% (SI 3.1, Figure S6). As the size of the UV-Vis beam is *ca*. 1.5 × 1 mm, while a significant proportion of the beam transmits through the 360 μm wide slots, BDD material is also encountered. This leads to some loss of light through absorption, scattering, and reflection. However, this value is still the highest reported value for a BDD OTE.

**Figure 2:**
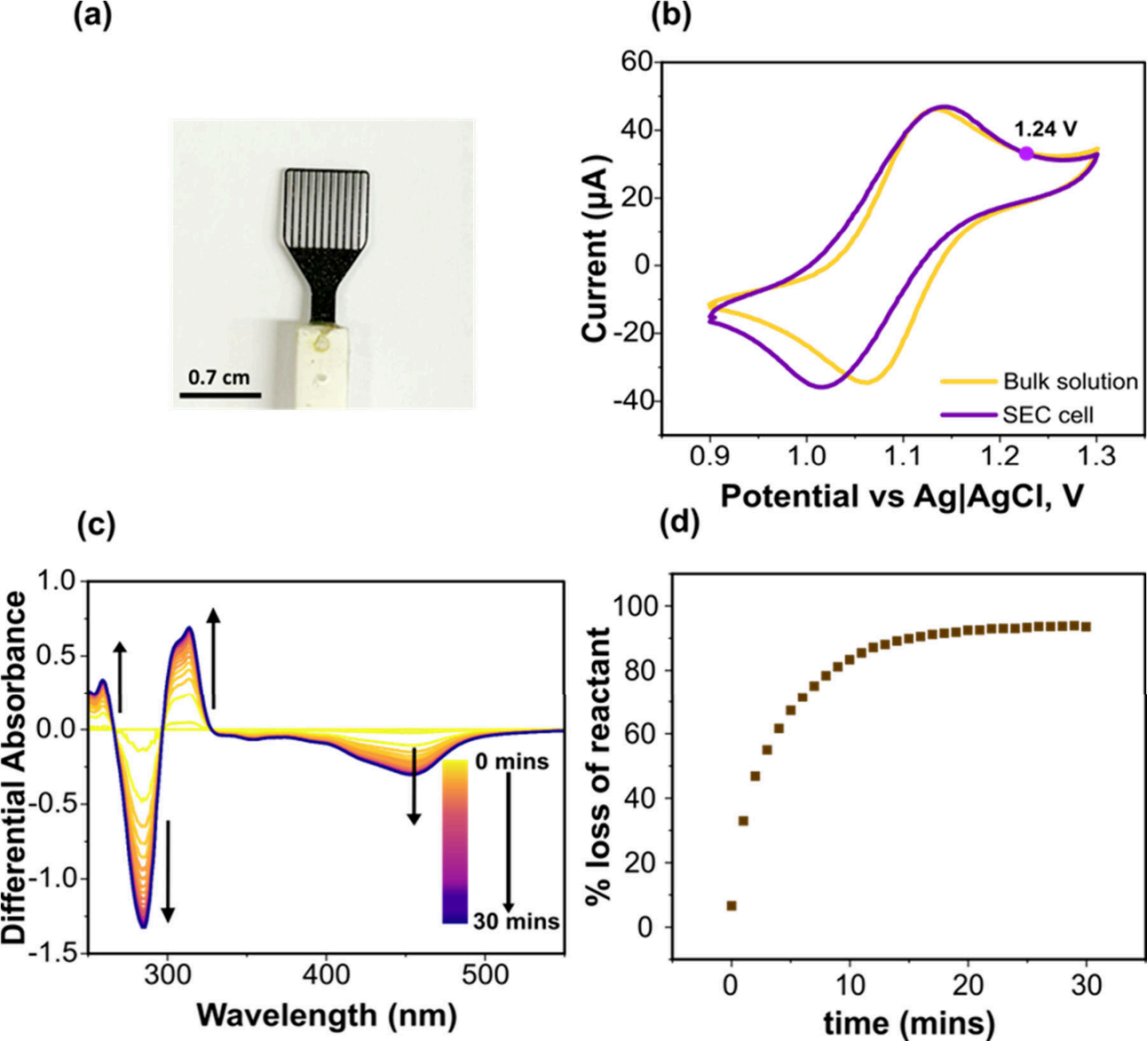
(a) Photograph of the BDD_SEC_ slot electrode. (b) CV response (third scan) of 0.25 mM Ru­(bipy)_3_^2+^ in 0.1 M KNO_3_ measured in the bulk solution and in the SEC cell at a scan rate of 0.1 V s^–1^ (indicated potential is the potential chosen for Ru­(bipy)_3_^2+^ oxidation for the measurement in (c). (c) UV-Vis SEC differential absorbance spectra for the oxidation of 0.25 mM Ru­(bipy)_3_^2+^ in 0.1 M KNO_3_ with the electrode held at +1.24 V vs Ag|AgCl (3 M Cl^–^). (d) % loss of reactant (Ru­(bipy)_3_^2+^) measured at 454 nm.

Initial experiments in the UV-Vis SEC cell focused on examining the electrochemical response of the electrode toward oxidation of Ru­(bipy)_3_^2+^ to Ru­(bipy)_3_^3+^ in a solution containing 0.25 mM Ru­(bipy)_3_^2+^ in 0.1 M KNO_3_. Ru­(bipy)_3_^2+/3+^ is a fast electron transfer redox couple, with a high positive *E*° = 1.26 V *vs* NHE.^33^ Its use also highlights the advantages of using BDD. The electrochemical response for Ru­(bipy)_3_^2+/3^ can be partially masked on more catalytically active electrodes due to its occurrence closer to the water oxidation window and/or in the region of surface oxidation for metals. This is demonstrated in SI 3.2, Figure S7, with CVs shown for the same solution of 0.25 mM Ru­(bipy)_3_^2+^ at BDD, Au, and glassy carbon (GC) disc electrodes in a bulk solution, *i.e.*, not confined within the small volume cell.

CVs recorded in the SEC cell and in bulk solution are shown in Figure 2b. The impact of the ohmic drop due to the small volume of solution on either side of the 400 μm thick BDD_SEC_ electrode in the 1 mm path length cuvette is evident. In the SEC cell a peak-to-peak separation (Δ*E*_*p*_) = 129 mV is recorded (Figure 2b, purple line), which compares with Δ*E*_*p*_ = 67 mV for the BDD_SEC_ electrode placed in bulk solution (Figure 2b, yellow line).^34^ Similar responses were observed when using the same electrode with a different fast electron transfer redox couple, FcTMA^+/2+^, at the same concentration, in the SEC cell (Δ*E*_*p*_ = 120 mV) and in bulk solution (Δ*E*_*p*_ = 70 mV) as shown in SI 2, Figure S5c.

For UV-Vis SEC experiments, the electrode was held at a potential to drive the oxidation of 0.25 mM Ru­(bipy)_3_^2+^ in 0.1 M KNO_3_ to Ru­(bipy)_3_^3+^ at a reasonably fast rate, here +0.10 V more positive than the forward peak potential in Figure 2b, *i.e.*, 1.24 V *vs* Ag|AgCl (3 M Cl^–^). Figure 2c shows continuously acquired UV-Vis differential absorption spectra over the wavelength range 250–550 nm for 30 min. Spectra are collected every minute, with each spectrum taking 3.75 s to record. Differential absorption spectra are obtained by subtracting the initial spectrum for the starting solution with no potential applied from that collected under potential control. The differential spectrum at 0 min corresponds to that recorded immediately after the application of the potential. A positive differential absorbance indicates the formation of electrochemically generated products, while a negative differential absorbance indicates depletion of reactants.

The differential absorbances at 287 and 454 nm, which correspond to the UV-Vis bands of the reactant Ru­(bipy)_3_^2+^, become more negative with time, associated with a decrease in the concentration of Ru­(bipy)_3_^2+^. In contrast, the differential absorption peaks at 258 and 315 nm, associated with the UV-Vis bands of Ru­(bipy)_3_^3+^, become more positive, attributed to an increase in Ru­(bipy)_3_^3+^ concentration. Isosbestic points are observed at 268, 298, and 333 nm which is evidence for an oxidation process that involves only two species, Ru­(bipy)_3_^2+^ and Ru­(bipy)_3_^3+^.^35^ This data also highlights one of the advantages of using the BDD_SEC_ electrode compared to ITO, in that the usable wavelength range can be extended below 300 nm and absorptions clearly distinguished from the background.^36^

The rate of loss of Ru­(bipy)_3_^2+^ as a function of time is calculated using eq 1,

1 $$\%\;loss\;of\;reac\tan t=\frac{A_{0}-A_{\left(t\right)}}{A_{0}}\times 100$$

where *A*_*0*_ is the absorbance of the reactant for a defined wavelength under no potential application (background subtracted with respect to that of the supporting electrolyte) and *A*_*t*_ is the absorbance at time, *t*. Figure 2d shows the % loss of Ru­(bipy)_3_^2+^ calculated at 454 nm (which corresponds to the ligand metal charge transfer – LMCT peak) as a function of time, reaching *ca*. 95% removal in *ca*. 20 min. *n* = 3 responses were shown to be reproducible as shown in Figure S8, SI 3.3. The observed experimental time scale is reflective of the fact the cell is not truly thin layer, due to the BDD thickness, diffusion length scale within the slots, and path length of the cell.^37^

### Dye Removal Using Electrochemical Advanced Oxidation Monitored *In Situ* via UV-Vis Spectroscopy

Electrochemical advanced oxidation processes have been used to remove organic contaminants, including synthetic dyes,^38^ from water, via breakdown to carbon dioxide, water, and other harmless products.^39,40^ BDD is thought to be particularly effective at indirect oxidation due to the proposed formation of (weakly absorbing) hydroxyl radicals (•OH),^13,41–43^ from water oxidation, provided sp^2^ carbon content is low.^44^ Indirect evidence for •OH formation from water oxidation on this free-standing BDD material (without holes) has been previously made using electron paramagnetic resonance spectroscopy, in combination with radical spin-traps.^45^ However, this work has the caveat that the spin trap can also be oxidized during the electrochemical advanced oxidation process.^45^

The efficiency of dye breakdown is commonly assessed via UV-Vis spectroscopy,^46^ where the decrease in dye concentration is measured as a function of time. Measurements are typically conducted *ex situ*, by taking aliquots. Acquiring *ex situ* measurements can be labor-intensive and time-consuming but also carries the risk of misinterpreting electrochemical data attributed to potential alterations in solution components during the transfer from the electrochemical cell to the spectroscopy set-up. Here we investigate the use of *in situ* UV-Vis SEC experiments to investigate the impact of electrode potential on the removal of BB FCF, a common blue dye pollutant.

Figure 3a shows the UV-Vis spectrum of 0.05 mM BB (in 1 M KNO_3_; pH = 6.88 ± 0.02) and the conjugated structure of the dye molecule. At this pH, BB can exist in two forms that remain in dynamic equilibrium. Three absorption maxima are visible at 308, 409, and 630 nm. The peaks at 308 and 409 nm are due to the auxochromes, and the latter peak corresponds to the maximum absorbance peak, *λ*_*max*_, which is due to the blue color-contributing component of the dye (chromophore).^47,48^ Figure 3b shows a CV of 0.05 mM BB in 1 M KNO_3_ recorded in the SEC cell using the BDD_SEC_ electrode by scanning oxidatively from 0 to 1.45 V and then reductively to −0.95 V *vs* Ag|AgCl (3 M Cl^–^) at a scan rate of 0.1 V s^–1^. Two irreversible anodic peaks are observed at 0.94 and 1.29 V *vs* Ag|AgCl (3 M Cl^–^), the origins of which have not been discussed in previous literature.

**Figure 3:**
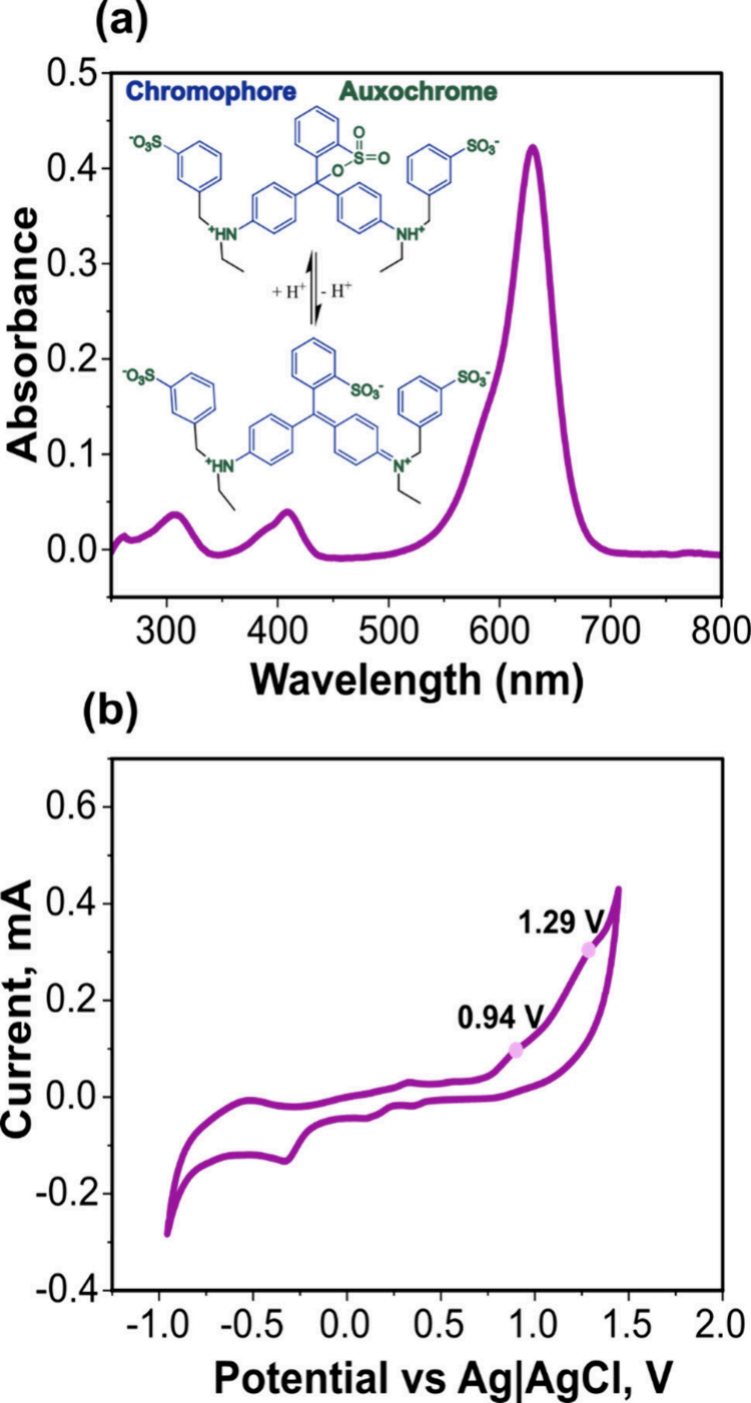
(a) UV-Vis spectrum of 0.05 mM BB in 1 M KNO_3_ (inset: BB structure) and (b) CV (third scan) of 0.05 mM BB in 1 M KNO_3_ scanned oxidatively at 0.1 V s^–1^ using the BDD_SEC_ slot electrode in the SEC cell.

For *operando* UV-Vis analysis of BB degradation in the SEC cell three oxidation potentials were chosen; 1.04, 1.39, and 2.22 V *vs* Ag|AgCl (3 M Cl^–^). The first two are *ca*. 0.1 V more positive than the two anodic peaks for BB oxidation and represent direct oxidation potentials. The third is into the water oxidation window on BDD and is chosen to be ∼0.1 V more positive than the thermodynamic potential for •OH generation from water oxidation, 2.12 V *vs* Ag|AgCl (3 M Cl^–^), at the measured solution pH (see SI 4.1);^49^ an indirect oxidation potential. The differential absorbance spectra (for 0.05 mM BB in 1 M KNO_3_) with the BDD_SEC_ electrode held at oxidizing potentials of 1.04, 1.39, and 2.22 V *vs* Ag|AgCl (3 M Cl^–^) are shown in Figures 4a, b, and c, respectively. UV-Vis measurements were recorded in the range 250–800 nm every 60 s for 30 mins during oxidation at 1.04 and 1.39 V *vs* Ag|AgCl (3 M Cl^–^) and every 30 s for 10 mins during oxidation at 2.22 V *vs* Ag|AgCl (3 M Cl^–^). Figure 4d shows the % BB color removal (at λ_max_ = 630 nm) as a function of time, calculated by using eq 1.

**Figure 4:**
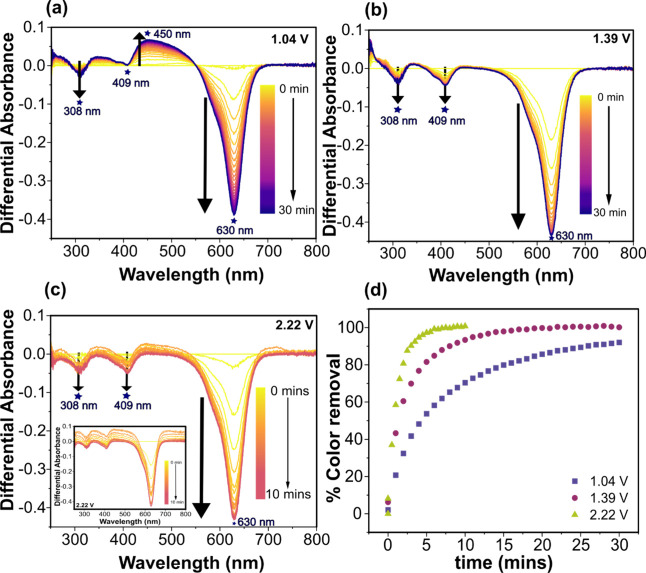
UV-Vis SEC differential absorbance spectra recorded for the oxidation of 0.05 mM BB in 1 M KNO_3_ on BDD_SEC_ slot electrode at (a) 1.04 V, (b) 1.39 V, and (c) 2.22 V vs Ag|AgCl (3 M Cl^–^): baseline adjusted (inset: no baseline adjustment) and (d) color removal % at 630 nm for different oxidation potentials.

At all potentials, the differential absorbance at λ_max_ = 630 nm becomes more negative with time, indicating dye discoloration. When a potential of 1.04 V *vs* Ag|AgCl (3 M Cl^–^) is applied, a gradual discoloration is observed, resulting in 92% color removal over 30 mins (at λ_max_). Interestingly, we observe a positive differential peak signal at 450 nm in the visible region of the spectrum growing with time, suggesting the degradation of BB into a smaller conjugated (UV-Vis active) structure (Figure 4a).^48^ The negative differential peak at 308 nm for BB becomes more negative with time, while we observe no change in the peak at 409 nm. This suggests that BB has degraded to a product that still retains a part of its original structure, which is UV-Vis active at 450 nm.^48^ By moving to the higher positive potential of 1.39 V *vs* Ag|AgCl (3 M Cl^–^), 100% colour removal is observed within 22 mins (for λ_max_). Under these conditions, no evidence of positive differential absorbance peaks (Figure 4b) are present, indicating that BB has degraded to UV-Vis inactive products (within the detection limits of the instrument). Both the differential auxochrome peaks at 308 and 409 nm also increase negatively with time. At the highest potential of 2.22 V *vs* Ag|AgCl (3 M Cl^–^), where •OH production is expected, 100% colour removal of BB (for λ_max_) is achieved within 6 mins. No positive differential absorbance peaks are observed. The baseline does shift slightly with time, most likely due to electrogenerated gas bubbles scattering the UV-Vis beam (Figure 4c). These could be a result of carbon dioxide evolution due to the complete mineralisation of the BB^50^ or oxygen evolution on defected (or sp^2^ carbon containing) areas of the BDD promoting alternative water oxidation routes.^51^ At potentials of 1.04 and 1.39 V *vs* Ag|AgCl (3 M Cl^–^), current densities (based on a BDD_SEC_ electrode geometric area of 1.35 cm^2^ and currents of ∼0.1 mA and 0.32 mA) of 7 × 10^–5^ A cm^–2^ and 2.4 × 10^–4^ A cm^–2^ result, increasing as the potential is extended to 2.2 V (current not measured). Quantitative measurements of BDD corrosion using the same grade of free-standing BDD material as used herein, show that even with a current density orders of magnitude higher (1 A cm^–2^), corrosion rates of <1 nm h^–1^ result.^29^ Significant increases in this value are only seen in the presence of very high concentrations of specific organic molecules such as 1 M acetic acid.^29^ Hence for these experiments herein, which use 5 × 10^–5^ M BB in a nitrate electrolyte at much lower current densities, for short time periods, we do not expect corrosion of the electrode surface to be an issue for consideration.

Assuming pseudo-first order kinetics for BB degradation, rate constants (*k*) were determined by taking the negative of the slope of the graph that plots the natural logarithm of BB absorbance at 630 nm as a function of time, as shown in SI 4.2, Fig S9. For applied potentials of 1.04, 1.39, and 2.22 V *vs* Ag|AgCl (3 M Cl^–^), rate constants of 0.10 min^–1^ (R^2^ = 0.980, for number of data points, n = 16), 0.24 min^–1^ (R^2^ = 0.990, n = 16),and 0.68 min^–1^ (R^2^ = 0.993, n = 11) were determined, respectively. The rate of color removal is significantly faster at the potential where both indirect and direct oxidation can occur. To prove complete mineralization at the highest potential, complementary follow-up experiments, such as total organic carbon (TOC), would be required, outside the scope of this paper.

### Monitoring Electrochemical Removal Rates of Metal Complexes in Aqueous and Nonaqueous Solutions

Electrodeposition is a commonly used technique to plate metals onto electrode surfaces from the metal salt solution via the application of a suitable potential (or current) to promote metal ion reduction.^52,53^ Electrodeposition is therefore used as a means of recovering metal ions from solution. Recently, there have been studies to investigate electrodeposition as a means of recovering metal catalysts from pharmaceutical product synthesis solutions.^54^

In order to determine removal rates and efficiency with respect to applied potential/current, electrolyte conditions, *etc.*, the loss of metal ions from solution is typically monitored using expensive inductively coupled plasma (ICP)–optical emission spectroscopy–mass spectrometry (MS) techniques, where aliquots are taken at various time points in the process.^55,56^ In this study, we investigate the use of UV-Vis SEC as an alternative (lower cost) *operando* technique under time scales where metal deposition does not cause a loss in optical transmission. BDD is a useful electrode material for metal recovery studies due to its mechanical durability and corrosion resistance in acid or alkali solutions. Electrodeposited metal on the BDD can be easily recovered using mechanical means or by chemical dissolution of the metal using appropriate solutions that leave the BDD electrode surface intact.

For these proof-of-concept studies, the metal complex, palladium (Pd) acetate (Pd_3_(OAc)_6_ in solid form), a rigid bidentate complex was employed, which is commonly used as a catalyst in organic Suzuki reactions.^57^ Experiments were first carried out using 1 mM Pd acetate in 0.05 M KCl in 0.1 M HCl;^54^ this concentration of Pd acetate is typical of that used in synthetic Suzuki reactions.^54^ An applied deposition potential (*E*_*dep*_) for the reduction of Pd acetate was determined by recording a CV in the SEC cell (SI 5.1, Figure S10b). The peak at −0.22 V *vs* Ag|AgCl (3 M Cl^–^) corresponds to the 2e^–^ reduction of Pd^2+^ ions to Pd. An *E*_*dep*_ of −0.33 V *vs* Ag|AgCl (3 M Cl^–^), *i.e.*, 0.1 V beyond the peak potential, was chosen for electrodeposition over a period of 30 mins.

The UV-Vis spectrum of the starting solution shows a broad low-intensity band at 450 nm associated with d–d transitions and intense bands at 222 and 278 nm associated with LMCT transitions arising from co-ordination of water molecules to Pd^2+^ (see SI 5.1, Figure S11).^58^ The acetate ligands in aqueous acid undergo hydrolysis resulting in a change of the inner coordination shell of Pd^2+^ ions from acetate ligands to water molecules.^59^ UV-Vis measurements were recorded simultaneously every 60 s, for 30 min, over the range 200–600 nm, Figure 5a. On reduction, the differential absorbance of all three bands can be seen to become more negative over 30 min, and importantly, no new positive peaks are observed. A positive peak would correspond to Pd(0) acetate solution species if reduction occurred without electrodeposition. This data support electrochemical removal of the Pd acetate from solution via electrodeposition. Figure 5b shows that 97% removal (eq 1, λ_max_ = 222 nm) is attained within 5 min at this potential. The SEM image in SI 5.2, Figure S12, shows that over 30 min, Pd electrodeposition has resulted only in the growth of isolated small submicrometer sized particles, which are not sufficient in size to impact light transmission through the slots.

**Figure 5:**
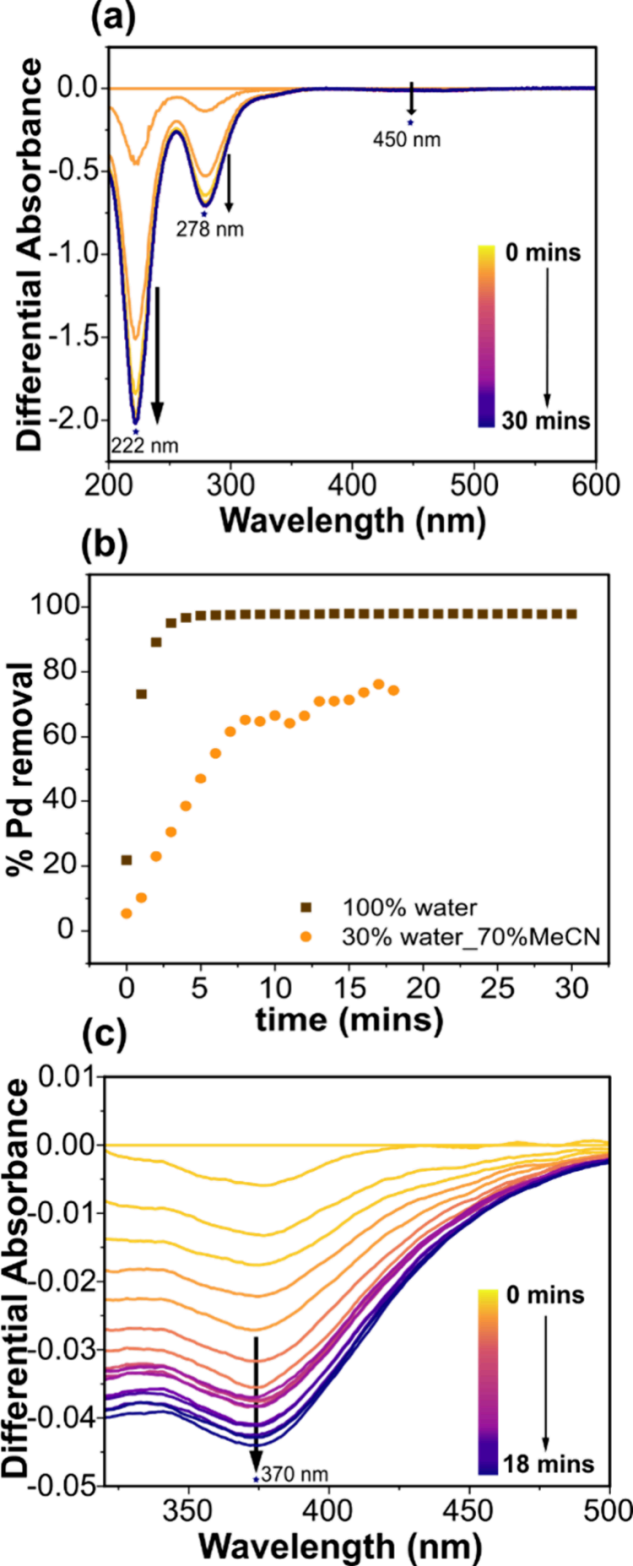
UV-Vis SEC differential absorbance spectra (a) reduction of 1 mM Pd acetate in 0.05 M KCl in 0.1 M HCl at −0.33 V vs Ag|AgCl (3 M Cl^–^), (b) % Pd removal in the aqueous acidic system and mixed solvent system, and (c) reduction of 1 mM Pd acetate in 0.1 M TBAPF_6_ in 30%:70% water:MeCN at −1.65 V vs Ag|Ag^+^.

Metal catalyst removal from organic solvents using electrodeposition is more challenging due to the ligands not necessarily undergoing solvolysis and preferring to remain bound to the metal during reduction.^41^ For this reason, in the case of electro-reductive removal of Pd acetate from MeCN, it was necessary to add a low percentage (%) of water to the MeCN to facilitate electrodeposition.^54^ To determine whether this concept could be verified using *operando* UV-Vis SEC, 1 mM Pd acetate in the mixed solvent system, water (30%):MeCN (70%) (v/v%), was investigated in a non-aqueous solvent compatible 3D printed SEC cell. An *E*_*dep*_ of −1.65 V *vs* Ag|Ag^+^, *i.e.*, 0.1 V past the peak potential, based on the CV obtained in the SEC cell for this mixed solvent system (SI 5.3, Figure S13), was employed. UV-Vis measurements were recorded simultaneously every minute in the range of 300-600 nm for 18 min (Figure 5c). A UV-Vis spectrum recorded in the mixed solvent solution (no potential) shows a peak at 370 nm for Pd acetate (see SI 5.3, Figure S14). In MeCN, only one (broad) peak at 400 nm is observed.^54^ On addition of water, the inner coordination shell of Pd ions changes from acetate to MeCN (and water), resulting in a blue shift in the UV-Vis spectra (in the region of 400 nm).^54^

Upon electrochemical reduction of Pd acetate in the mixed solvent, the differential absorption peak at 370 nm becomes more negative over time, and importantly there are no positive peaks appearing (associated with reduction to a ligated Pd(0) complex, which remains in solution), Figure 5c. The differential absorbance values for the mixed solvent system are lower than the aqueous acid system, due to the d–d transition (monitored here) having a significantly lower extinction coefficient than the LMCT band monitored in the aqueous system. This data supports previous *ex situ* verifications of electrodeposition of Pd from Pd acetate in the mixed solvent system using electron microscopy and Pd ICP-MS.^54^ Figure 5b shows the % Pd removal from the mixed solvent system calculated for *λ*_*max*_ = 370 nm using eq 1. Assuming pseudo-first order kinetics, the rate constants of Pd removal from the aqueous acidic system and mixed solvent system were determined as shown in SI 5.4, Figure S15. The rate constant, k = 0.09 min^–1^ (R^2^ = 0.997, n = 6) for Pd removal from the mixed solvent system was an order of magnitude lower than Pd removal from the aqueous acidic system where k = 0.92 min^–1^ (R^2^ = 0.995, n = 4). This data quantitatively show that it is easier for Pd acetate to undergo ligand exchange which facilitates electrodeposition in an aqueous system than in an aqueous–nonaqueous miscible solution.

## Conclusion

While the concept of using a BDD free-standing electrode into which through-holes have been laser-machined to increase light transmission for combined UV-Vis SEC studies is not new, we describe the first applications that capitalize on the advantageous properties of BDD. In particular, we show the ability of the BDD_SEC_ UV-Vis system to comparatively assess the removal rates of a UV-active molecule as a function of applied oxidative potential, *operando*, in the simple setup described. While we apply the system to the dye, brilliant blue, the approach can be used with any advanced oxidation pollutant that shows a measurable UV-Vis signal. Rates for removal of the dye are extracted by making time dependent measurements. The pseudo-first order kinetic rate constant for brilliant blue removal, in this electrochemical cell, is shown to increase with applied potential: 0.10 min^–1^ (1.04 V), 0.24 min^–1^ (1.39 V), and 0.68 min^–1^ (2.22 V) *vs* Ag|AgCl (3 M Cl^–^). Identification of UV-active reaction intermediates during the breakdown process is also possible. For BDD we quantify that removal rates are significantly faster at potentials where we believe both direct and indirect oxidation of the dye is possible. While complementary measurements of, *e.g.*, TOC, would be needed to verify complete mineralization, the methodology provides a fast and efficient method for quantifying and comparing parent molecule removal rates as a function of applied potential (current).

We have also demonstrated that the UV-Vis SEC methodology is useful for *operando* assessment of metal ion removal rates in different solvent systems and distinguishing between reduction of the metal ion complex to either the electrodeposited metal or a soluble metal(0) complex. In particular, we demonstrate in both aqueous and mixed solvent solutions Pd acetate reduces to Pd metal. We determine that the rate constant for removal in an aqueous acidic solution is an order of magnitude faster than in the mixed water:acetonitrile system. Due to not wanting to compromise optical transmission, measurement time scales must be such that growth of metal over the slot holes is not promoted.

Finally, while it is possible to use inserts between the electrode and cuvette to reduce cell thickness as others have done to achieve thin layer cell behavior and decrease equilibration times,^23,27^ thinning of the free-standing BDD would also be required. Free-standing BDD as thin as ∼50 μm has been prepared previously;^60^ however, this material must be handled with care to avoid breakages. Finite element modeling would aid in determining the SEC capabilities of the BDD electrode in the SEC cell and the preferred through-hole arrangement for optimal performance.

## Supplementary Material



## Notes

Details on BDD_SEC_ electrode preparation and UV-Vis SEC set up; electrochemical characterization and optical transmittance of the BDD_SEC_ electrode; comparison of BDD with other electrode materials; data to prove reproducibility of the setup; rate of BB degradation and Pd removal; CV of Pd acetate in the aqueous acidic system and mixed solvent system; SEM of Pd electrodeposition on the BDD_SEC_ electrode (PDF)
