# Supplementary material for: Free-Standing Boron Doped Diamond Slot Electrodes for UV–Visible Spectroelectrochemistry: Electrochemical Advanced Oxidation and Metal Ion Reduction
Source: ACS Electrochem. 2025 May 12;1(8):1462–71. doi: 10.1021/acselectrochem.5c00085 (PMC12337086; doi:10.1021/acselectrochem.5c00085)
Supplement: Supplementary file 1 [file ec5c00085_si_001.pdf]

Supporting Information

Free-Standing Boron Doped Diamond Slot Electrodes For UV-Vis Spectroelectrochemistry : Electrochemical Advanced Oxidation and Metal Ion Reduction

Anjali John,[1] *Anna Dettlaff,*[2] *Joshua. J. Tully*[1] and *Julie. V. Macpherson*[1]*

[1]Department of Chemistry, University of Warwick, Coventry, UK, CV4 7AL
[2]Faculty of Chemistry, Gdańsk University of Technology, Narutowicza 11/12,80-233 Gdańsk, Poland

Table of Contents

SI 1: Electrode preparation and UV-Vis SEC set up

SI 1.1: BDD_{SEC} electrode fabrication

SI 1.2: SEC cell

SI 1.3: 3D printed platform

SI 1.4: BDD_{disc} electrode fabrication

SI 2: Electrochemical characterization of the BDD_{SEC} slot electrode

SI 3: Spectroelectrochemical characterization of the BDD_{SEC} slot electrode

SI 3.1: Optical transmittance

SI 3.2: Comparison with different electrode materials

SI 3.3: UV-Vis SEC oxidation of $Ru(bipy)_3^{2+}$ using BDD_{SEC} slot electrode: demonstration of reproducibility

SI 4: Dye removal using electrochemical advanced oxidation monitored in-situ via UV-Vis

SI 4.1: Thermodynamic potential for •OH generation calculation

SI 4.2: Rate of BB degradation

SI 5: Monitoring electrochemical removal of metal complexes indifferent solutions via electrodeposition using BDD_{SEC}

SI 5.1: Aqueous system of palladium acetate

SI 5.2: SEM of Pd electrodeposition on the BDD_{SEC} slot electrode

SI 5.3: Palladium acetate in mixed solvent system

SI 5.4: Rate of Pd removal

References

SI 1: Electrode preparation and UV-Vis SEC set-up

SI 1.1: BDD_{SEC} electrode fabrication

The laser-machined BDD_{SEC} electrodes were housed in 3D-printed rigid 10 K resin (Fig S1a). The steps involved in the integration of the electrodes into this housing are detailed below:

Step 1: Copper wire is glued in place in the electrode housing (Fig S1a right and Fig S1c) using super glue (Loctite, USA), the end of the Cu wire is flattened for contact.

Step 2: With the contact pad facing up, the SEC electrode is glued into the electrode housing (Fig S1a left) as shown in Fig S1b.

Step 3: Conductive epoxy (Chemtronics, USA) is applied on the contact pad and the two housings are pressed together.

Step 4: Rigid 10K resin is gently poured around the sides and over the top of the housing to seal the electrodes and UV-cured using a UV lamp for a few seconds until the resin has hardened

Step 5: Final UV curing takes place in a UV curing oven (Form Cure, FormLabs, USA) at 60°C for 60 minutes.

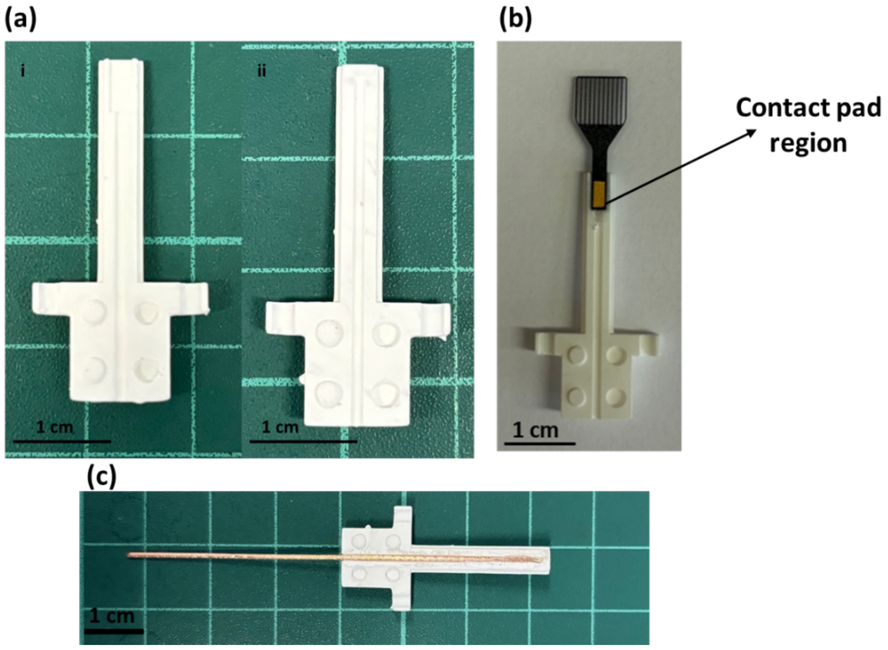

Figure S1. (a) 3D printed electrode housings, (i) for placing the lasered electrode and (ii) for placing copper wire, (b) SEC electrode glued onto electrode housing (Ti|Pt|Au ohmic contact sputtered onto the contact pad region), and (c) copper wire glued onto electrode housing

SI 1.2: SEC cell

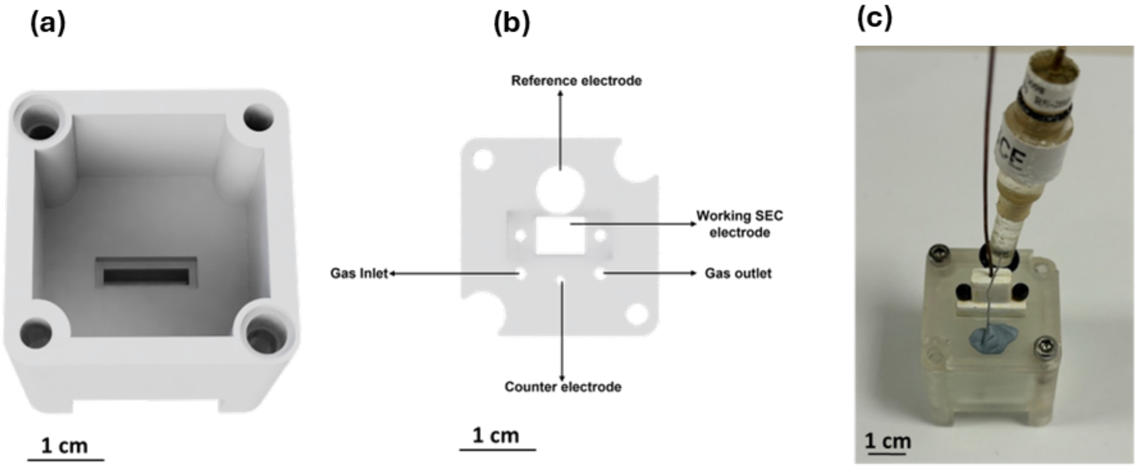

Figure S2. (a) 3D render of SEC cell body, (b) 3D render of SEC cell cap, and (c) top view of the SEC cell set up

SI 1.3: 3D printed platform

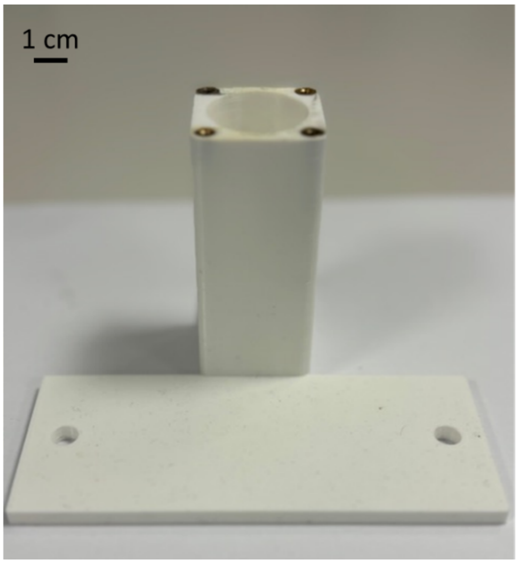

Figure S3. 3D printed platform for SEC cell mounting, using fused deposition modelling (FDM) in combination with PLA, on an Ultimaker S5 (Ultimaker, Netherlands)

SI 1.4: BDD_{disc} electrode fabrication

The electrode was fabricated using a procedure described elsewhere.[1] 1 mm BDD discs were laser machined from a freestanding, polished (~ 6 nm RMS roughness) wafer of electroanalytical grade (EA) BDD (electrode E in reference[2] Element Six Limited, UK) of 357 μm thickness using a 355 nm Nd:YAG 34 ns pulse laser micromachining system (E-355H-ATHI-0 system, Oxford Lasers). BDD discs were

acid cleaned by immersing in a boiling mixture of concentrated sulfuric acid (H_2SO_4, > 96%, Merck) and potassium nitrate (KNO_3, 99.97%, Sigma Aldrich) to minimise the sp^2 carbon content in the surface, resulting from laser micromachining. An ohmic contact was formed by sputtering Ti (10 nm) / Au (400 nm) (MiniLab 060, Moorfield, UK) onto the nucleation face of the BDD disc followed by annealing at 400°C for five hours. The BDD discs were sealed in glass capillaries (outer diameter 2 mm; inner diameter 1.16 mm; Harvard Apparatus Ltd., Kent, UK), with the BDD surface exposed by removing the glass by polishing with carbide grit paper of different grades starting with coarse to fine. Electrical contact was made to the sputtered face of the BDD disc using conductive epoxy and copper wire for an external electrical connection. The capillary was sealed with non-conducting epoxy (Araldite).

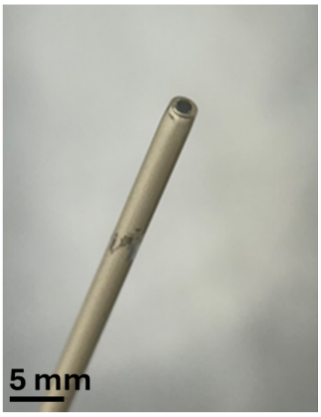

Figure S4. Photograph of 1 mm BDD disc electrode sealed in glass

SI 2: Electrochemical characterization of the BDD_{SEC} slot electrode

Table S1. Electrode characterisation data

SEC electrode	*A* (cm^2)	*C* (µF cm^{-2})	Solvent window (V)	ΔE_p measured in bulk (mV)	ΔE_p measured in SEC cell (mV)
BDD_{SEC} slot electrode	1.35	12.5	3.81	70	120

Electrochemical double layer capacitance, *C*, for the BDD_{SEC} slot electrode (Table S1) was determined at 0 V *vs* Ag|AgCl (3 M Cl^-) by scanning reductively from 0 V to -0.1 V to 0.1 V at a scan rate of 0.5 V s^{-1} in 0.1 M KNO_3 (Fig. S5a) using $C = i_{average} \times \nu / A$, where $i_{average}$ is the current average of the forward and reverse sweep, ν is the scan rate and *A* is the geometric electrode area. Aqueous solvent windows were recorded in 0.1 M KNO_3 at 0.1 V s^{-1} from -2 V to 2 V *vs* Ag|AgCl (3 M Cl^-) for three cycles (Fig. S5b). Anodic and cathodic potential limits are defined at a slightly lower current density of 0.3 mA cm^{-2} (rather than 0.4 mA cm^{-2}),[2] given within the potential window measured it was not possible to reach 0.4 mA cm^{-2}. sp^2 carbon presence on the BDD_{SEC} electrode (due to laser machining and exposure of the nucleation face of the as-grown BDD_{SEC} electrode to solution), resulted in a visible oxygen reduction peak *ca*. -0.7 V vs Ag|AgCl (3 M Cl^-) in the cathodic window. On the anodic window, an oxidation peak around 1.0 V *vs* Ag|AgCl (3 M Cl^-), thought to be due to oxidation of sp^2 carbon is observed.[3]

To investigate the impact of uncompensated resistance in the SEC cell, CV responses for the outer sphere redox mediator, ferrocenylmethyltrimethylammonium ($FcTMA^+$) in a beaker (bulk solution) and in the SEC cell were recorded. The solution comprised 0.25 mM $FcTMA^+$ in 0.1 M KNO_3 and scanned oxidatively from 0.2 V to 0.7 V vs Ag|AgCl (3 M Cl^-). Three CV cycles were recorded at 0.1 V s^{-1} (Fig. S5c). ΔE_p obtained in bulk is very close to what is expected for a electrochemically reversible redox couple. The values of ΔE_p recorded in the SEC cell were higher than when measured in the bulk solution (Table S1). This is attributed to the solution resistance resulting from working in a comparatively thin layer of solution surrounding the SEC electrode along with the contribution of the large current resulting from the large electrode area.[4]

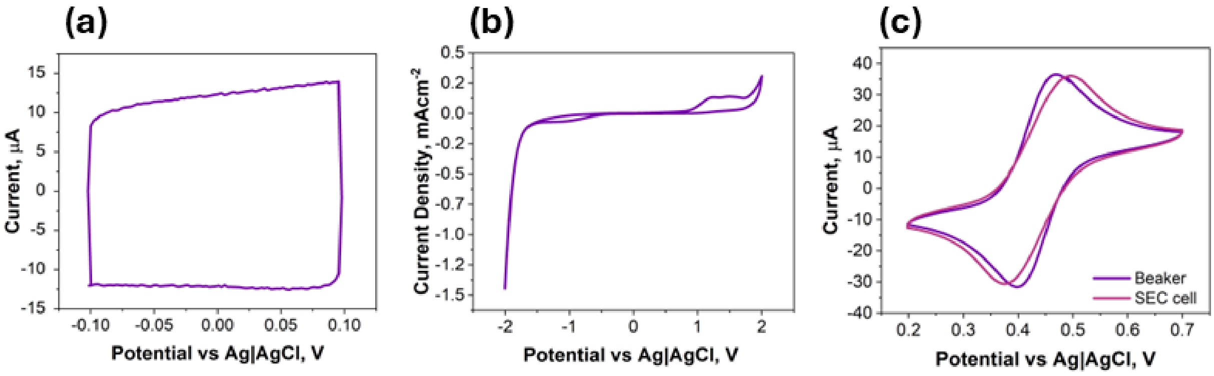

Figure S5. Electrode characterisation measurements on a BDD_{SEC} slot electrode: (a) Capacitance CV (third scan) electrode in 0.1 M KNO_3 at 0.5 V s^{-1}, (b) solvent window CV (third scan) recorded in 0.1 0.1 M KNO_3 at 0.1 V s^{-1}, and (c) CV (third scan) recorded in 0.25 mM $FCTMA^+$ in 0.1 M KNO_3 at 0.1 V s^{-1} in bulk solution and the SEC cell.

SI 3: Spectroelectrochemical characterization of the BDD_{SEC} slot electrode

SI 3.1 Optical transmittance

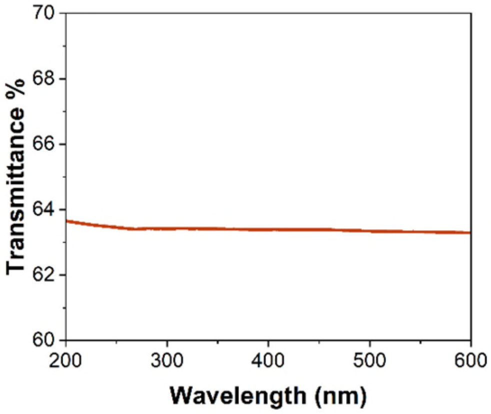

Figure S6. Optical transmittance of the BDD_{SEC} slot electrode in air

SI 3.2 Comparison of different electrode materials

On the BDD electrode, the CV is as expected for a fast electron transfer redox couple, with a ΔE_p of 65 mV. For both glassy carbon (GC) and Au, the redox peaks are slightly masked due to higher non-faradaic current contributions and proximity to the water oxidation solvent window. Additionally, the Au electrode undergoes self-oxidation during the $Ru(bipy)_3^{2+}$ oxidation.[4] Given the extended aqueous anodic window for indium tin oxide, this OTE shows a response similar to BDD.[5,6]

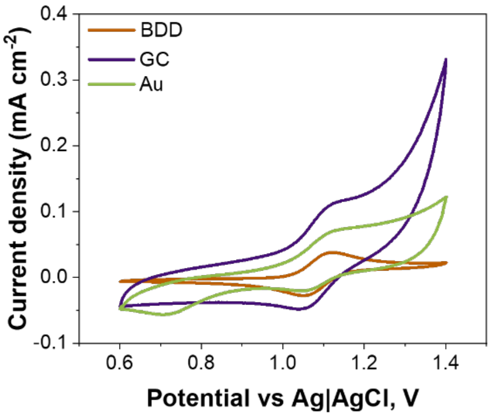

Figure S7. CV response (third scan) for 0.25 mM $Ru(bipy)_3^{2+}$in 0.1 M KNO_3 recorded at a 1 mm diameter BDD disc electrode, 2.5 mm diameter Au disc electrode and 3 mm diameter GC disc electrode at 0.1 V s^{-1}

SI 3.3 UV-Vis SEC oxidation of $Ru(bipy)_3^{2+}$ using BDD_{SEC} slot electrode: demonstration of reproducibility

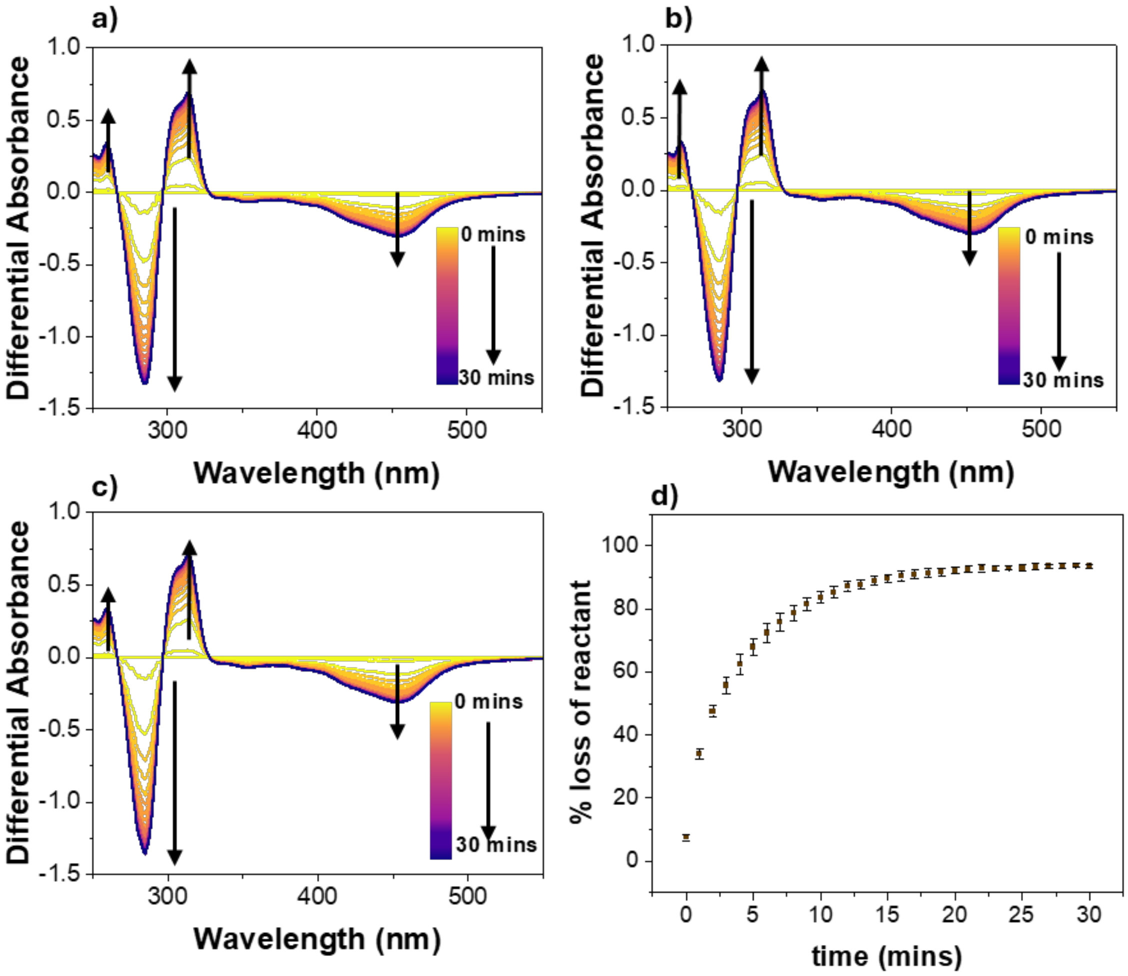

Figure S8. (a,b,c) UV-Vis spectrum obtained for the electrochemical oxidation of 0.25 mM $Ru(bipy)_3^{2+}$ in 0.1 M KNO_3 for n=3 at 1.24 V *vs* Ag|AgCl (3 M Cl^-) using the BDD_{SEC} slot electrode, (d) mean % loss of reactant (n=3) with error bars.

SI 4: Dye removal using electrochemical advanced oxidation monitored *in-situ* via UV-Vis spectroscopy

SI 4.1 Thermodynamic potential for •OH generation calculations

The standard thermodynamic potential for hydroxyl radical generation on BDD according to reaction (S1) is E° = 2.73 V *vs* RHE. [7,8]

$$H_2O \rightarrow \bullet OH + H^+ + e^- \qquad E^{\circ} = 2.73\ V\ vs\ RHE \qquad (S1)$$

The thermodynamic potential for hydroxyl radical production in the solution of 0.05 mM BB FCF with 1 M KNO_3 (pH = 6.88 ± 0.02) was calculated based on Eq. (S2) and it is equal to 2.12 V vs Ag|AgCl (3M Cl^-).

$$E_{Ag|AgCl}\,(V) = E^{\circ}_{RHE}\,(V) - (0.059 \times pH) - E^{\circ}_{Ag|AgCl}(V) \qquad (S2)$$

SI 4.2 Rate of BB degradation

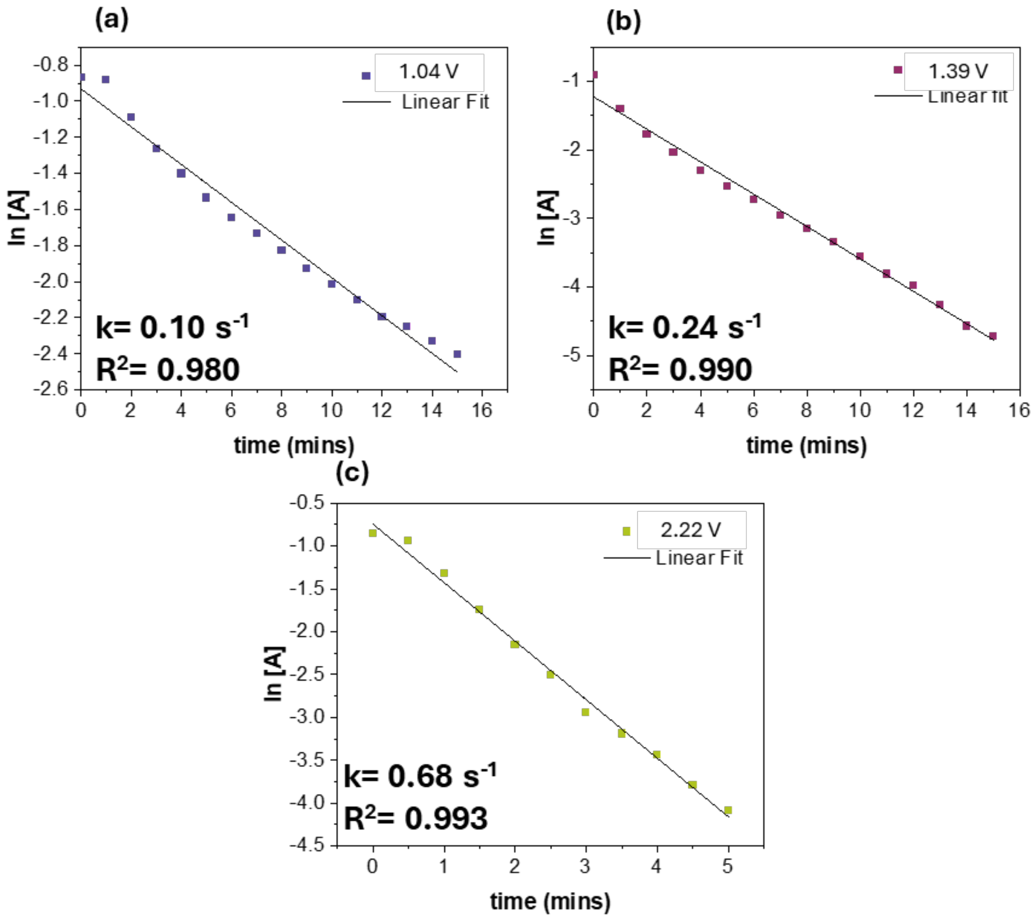

Figure S9. Fitting of experimental data to a kinetic model of first-order reaction kinetics for BB degradation at (a) 1.04 V *vs* Ag|AgCl (3 M Cl^-), (b) 1.39 V *vs* Ag|AgCl (3 M Cl^-) and (c) 2.22 V *vs* Ag|AgCl (3 M Cl^-).

SI 5: Monitoring electrochemical removal rates of metal complexes in aqueous and non-aqueous solutions

SI 5.1 Aqueous system of palladium acetate

To understand the electrochemical behavior of palladium (Pd) acetate in an aqueous environment, CV studies were conducted using a BDD_{SEC} slot electrode in a conventional glass cell as shown in Figure S10 a. The CV was recorded by scanning reductively initially between -0.4 V and 1.2 V *vs* Ag|AgCl (3 M Cl^-). Scanning in the negative direction, the peak number labelled 1 indicates a two-electron reduction of Pd(II) to Pd (0), and peak number 2 corresponds to H adsorption on the electrodeposited Pd and the hydrogen evolution reaction. On the reverse scan, peak number 3 corresponds to H desorption and 4 corresponds to stripping of electrodeposited Pd.[9,10] The applied potential for the reduction of Pd acetate for the SEC studies was determined by recording a CV and first scanning reductively between -0.4 V and 1.2 V *vs* Ag|AgCl (3 M Cl^-) in the SEC cell (Figure S10b).

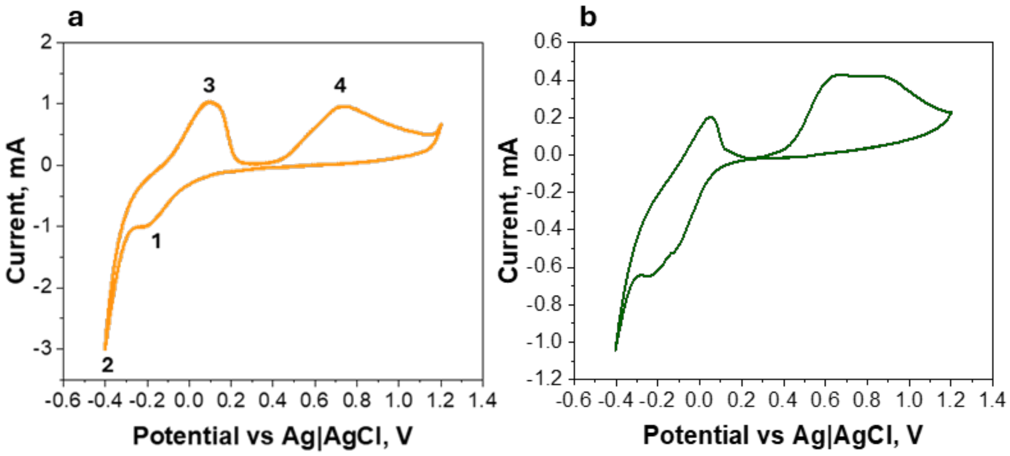

Figure S10. (a) CV of 1 mM Pd acetate in 0.05 M KCl in 0.1 M HCl purged under N_2 scanned reductively scan rate of 0.1 V s^{-1} using the BDD_{SEC} slot electrode in a conventional glass cell (third scan) and (b) the SEC cell (third scan) respectively.

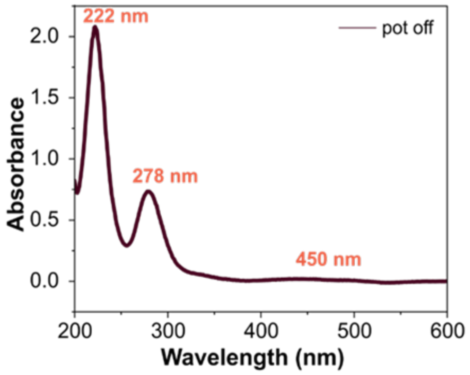

Figure S11. UV-Vis of 1 mM Pd acetate in 0.05 M KCl in 0.1 M HCl before application of reduction potential (absorbance subtracted from the absorbance of 0.05 M HCl + 0.1 M KCl)

SI 5.2 SEM of Pd electrodeposition on the BDD_{SEC} slot electrode

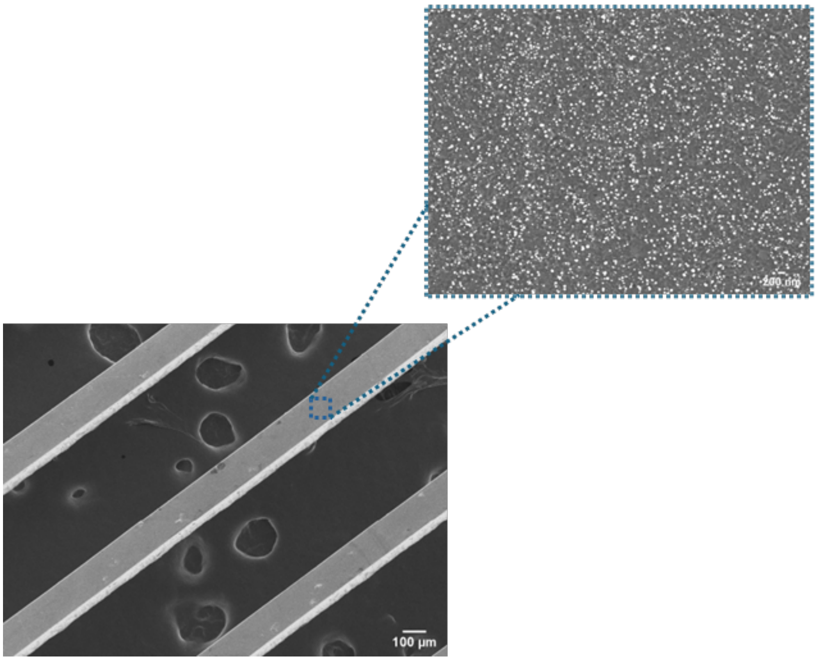

Figure S12. SEM at 150× magnification of the BDD_{SEC} slot electrode post 30 mins Pd electrodeposition from a solution of 1 mM Pd acetate in 0.05 M KCl in 0.1 M HCl at -0.33 V vs Ag|AgCl (3 M Cl^-). The electrode was placed on a carbon tape for SEM imaging. Inset: Pd nanoparticles formed during electrodeposition, image at 25k × magnification. Zeiss Gemini at 4 keV and a working distance of 6.4 mm using InLens detector.

SI 5.3 Palladium acetate in mixed solvent system

To choose the potential for Pd deposition from a solution of 1 mM Pd acetate in 0.1 M $TBAPF_6$ in 30% water : 70% MeCN (v/v%), CV was performed at a scan rate of 0.1 V s^{-1} in the SEC cell (Figure S13). On scanning reductively, two reduction peaks were observed at -0.85 V vs Ag|Ag^+ (peak number 1) and -1.55 V vs Ag|Ag^+ (peak number 2), followed by the onset of hydrogen evolution reaction at -1.8 V vs Ag|Ag^+. In this mixed solvent system the inner solvation of Pd can consist of water and MeCN molecules. The peak at -0.85 V vs Ag|Ag^+ is likely due to Pd^{2+} reduction where the Pd ions are primarily solvated by water molecules, whilst the peak at -1.55 V vs Ag|Ag^+ likely corresponds to the reduction of Pd^{2+} ions solvated by MeCN. [11] To facilitate Pd electrodeposition, a potential 0.1 V past -1.55 V vs Ag|Ag^+ was chosen.

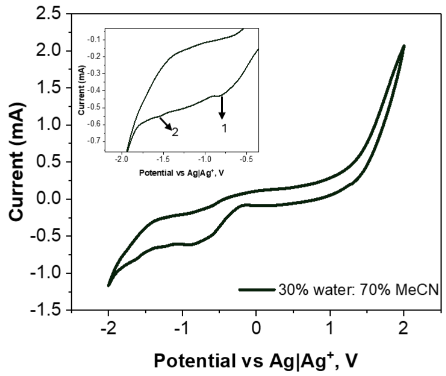

Figure S13. CV (third scan) of 1 mM Pd acetate in 0.1 M $TBAPF_6$ in 30% water : 70% MeCN (v/v%) scanned reductively using the BDD_{SEC} electrode in the SEC cell at a scan rate of 0.5 V s^{-1}. Inset: zoomed in CV between -0.5 V vs -2.0 V *vs* $Ag|Ag^+$.

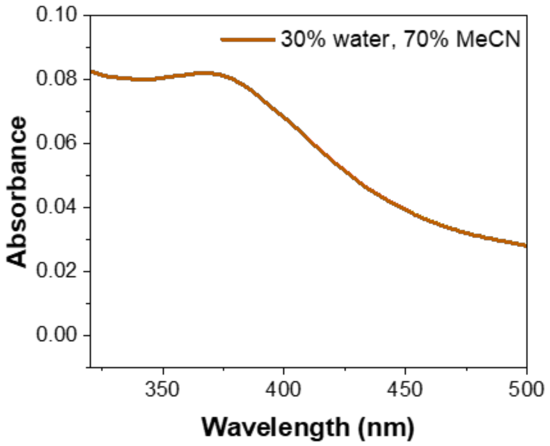

Figure S14. UV-Vis absorbance of the initial solution of 1 mM of Pd acetate in 0.1 M $TBAPF_6$ in 30% water : 70% MeCN.

SI 5.4 Rate of Pd removal

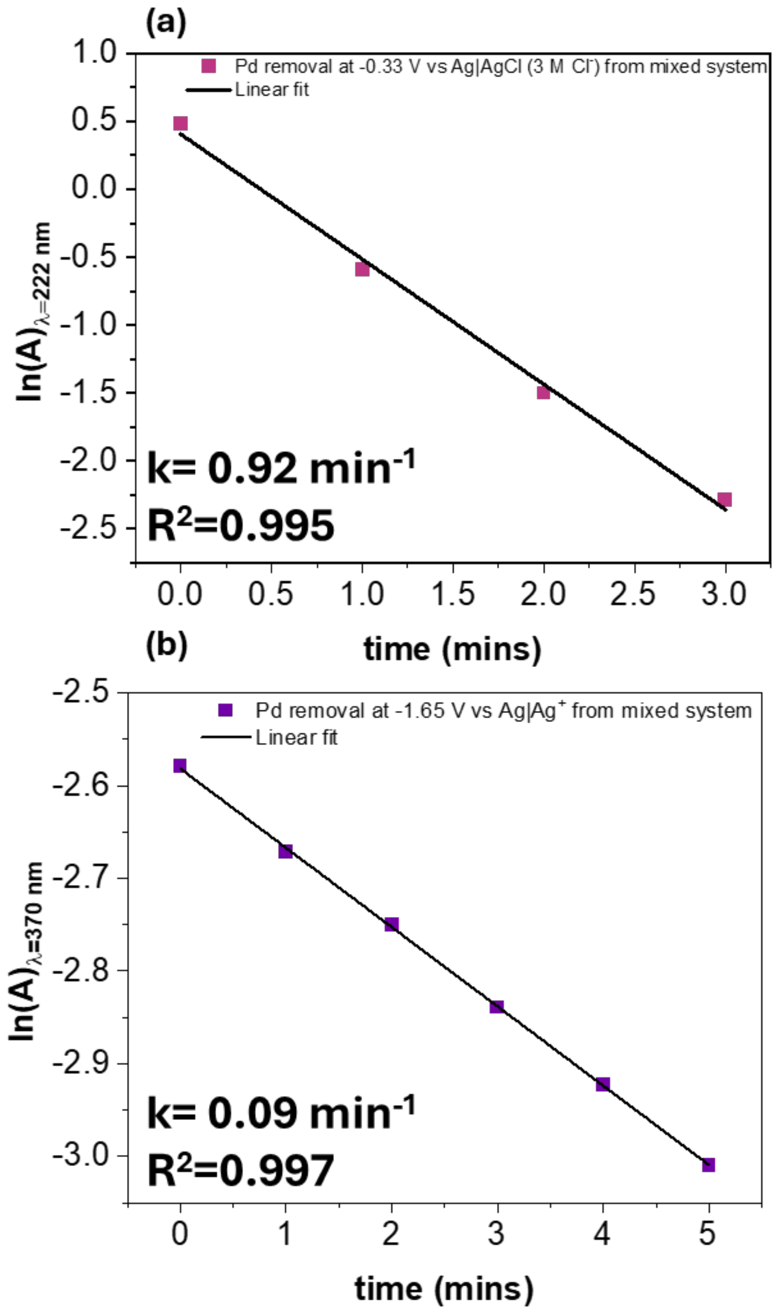

Figure S15. Fitting of experimental data to a kinetic model of pseudo-first order reactions of Pd removal (a) from a solution of 1 mM Pd Ac in 0.05 M KCl in 0.1 M HCl at -0.33 V vs Ag|AgCl (3 M Cl^-), and (b) from 1 mM Pd acetate in 0.1 M $TBAPF_6$ in 30% water : 70% MeCN (v/v%) at -1.65 V vs Ag|Ag^+.

REFERENCES

(1) Hutton, L., Newton, M. E. Unwin, P. R., Macpherson, J. V. Amperometric Oxygen Sensor Based on a Platinum Nanoparticle-Modified Polycrystalline Boron Doped Diamond Disk Electrode. *Anal Chem* **2009**, *81* (3), 1023–1032.

(2) Hutton, L. A., Iacobini, J. G., Bitziou, E., Channon, R. B., Newton, M. E.; Macpherson, J. V. Examination of the Factors Affecting the Electrochemical Performance of Oxygen-Terminated Polycrystalline Boron-Doped Diamond Electrodes. *Anal Chem* **2013**, *85* (15), 7230–7240.

(3) Show, Y., Witek, M. A., Sonthalia, P., Swain, G. M. Characterization and Electrochemical Responsiveness of Boron-Doped Nanocrystalline Diamond Thin-Film Electrodes. *Chem. Mater*. **2003**, *15* (4), 879–888.

(4) Cherevko, S., Topalov, A. A., Zeradjanin, A. R., Katsounaros, I., Mayrhofer, K. J. J., Gold Dissolution: Towards Understanding of Noble Metal Corrosion. *RSC Adv* **2013**, *3* (37), 16516–16527.

(5) Stotter, J.; Show, Y.; Wang, S.; Swain, G. Comparison of the Electrical, Optical, and Electrochemical Properties of Diamond and Indium Tin Oxide Thin-Film Electrodes. *Chem Mater.* **2005**, *17* (19), 4880–4888.

(6) Villemure, G.; Pinnavaia, T. J. Cyclic Voltammetry of Tris(2,2‘-Bipyridyl)Ruthenium(II) Cations Adsorbed in Electrodes Modified with Mesoporous Molecular Sieve Silicas. *Chem Mater.* **1999**, *11* (3), 789–794.

(7) Braxton, E., Fox, D. J., Breeze, B. G., Tully, J. J., Levey, K. J., Newton, M. E., Macpherson, J. V. Electron Paramagnetic Resonance for the Detection of Electrochemically Generated Hydroxyl Radicals: Issues Associated with Electrochemical Oxidation of the Spin Trap. *ACS meas. sci. au* **2023**, *3* (1), 21–31.

(8) Armstrong, D. A., Huie, R. E., Koppenol, W. H., Lymar, S. V., Merenyi, G., Neta, P., Ruscic, B., Stanbury, D. M., Steenken, S., Wardman, P. Standard Electrode Potentials Involving Radicals in Aqueous Solution: Inorganic Radicals (IUPAC Technical Report). *Pure Appl. Chem.* **2015**, *87* (11–12), 1139–1150.

(9) Hussein, H. E. M., Ray, A. D., Macpherson, J. V. Removal from a Palladium Acetate – Acetonitrile System via Trace Water Addition *Green Chem.* **2019**, 4662–4672.

(10) Wang, Y., Yuan, J., Wang, L., Hao, C. Electrochemical Study on Palladium Acetate + p-Benzoquinone + molybdovanadophosphate System by Cyclic Voltammetry. *J Chem Eng Data* **2009**, *55* (6), 2233–2237.

(11) Hussein, H. E. M.; Amari, H.; Breeze, B. G.; Beanland, R.; Macpherson, J. V. Controlling Palladium Morphology in Electrodeposition from Nanoparticles to Dendrites: Via the Use of Mixed Solvents. *Nanoscale*. **2020**, *12* (42), 21757–21769.
